# Valvular imaging in the era of feature‐tracking: A slice‐following cardiac MR sequence to measure mitral flow

**DOI:** 10.1002/jmri.26971

**Published:** 2019-10-25

**Authors:** Felicia Seemann, Einar Heiberg, Marcus Carlsson, Ricardo A. Gonzales, Lauren A. Baldassarre, Maolin Qiu, Dana C. Peters

**Affiliations:** ^1^ Department of Clinical Sciences Lund, Clinical Physiology Lund University, Skåne University Hospital Lund Sweden; ^2^ Department of Biomedical Engineering, Faculty of Engineering Lund University Lund Sweden; ^3^ Department of Radiology & Biomedical Imaging, Yale School of Medicine Yale University, New Haven Connecticut USA; ^4^ Wallenberg Center for Molecular Medicine Lund University Lund Sweden; ^5^ Department of Electrical Engineering Universidad de Ingenieria y Tecnologia Lima Peru; ^6^ Department of Cardiology, Yale School of Medicine Yale University New Haven Connecticut USA

**Keywords:** mitral valve flow, slice‐following, cardiovascular magnetic resonance, phase contrast, feature‐tracking

## Abstract

**Background:**

In mitral valve dysfunction, noninvasive measurement of transmitral blood flow is an important clinical examination. Flow imaging of the mitral valve, however, is challenging, since it moves in and out of the image plane during the cardiac cycle.

**Purpose:**

To more accurately measure mitral flow, a slice‐following MRI phase contrast sequence is proposed. This study aimed to implement such a sequence, validate its slice‐following functionality in a phantom and healthy subjects, and test its feasibility in patients with mitral valve dysfunction.

**Study Type:**

Prospective.

**Phantom and Subjects:**

The slice‐following functionality was validated in a cone‐shaped phantom by measuring the depicted slice radius. Sixteen healthy subjects and 10 mitral valve dysfunction patients were enrolled at two sites.

**Field Strength/Sequence:**

1.5T and 3T gradient echo cine phase contrast.

**Assessment:**

A single breath‐hold retrospectively gated sequence using offline feature‐tracking of the mitral valve was developed. Valve displacements were measured and imported to the scanner, allowing the slice position to change dynamically based on the cardiac phase. Mitral valve imaging was performed with slice‐following and static imaging planes. Validation was performed by comparing mitral stroke volume with planimetric and aortic stroke volume.

**Statistical Tests:**

Measurements were compared using linear regression, Pearson's R, parametric paired *t*‐tests, Bland–Altman analysis, and intraclass correlation coefficient (ICC).

**Results:**

Phantom experiments confirmed accurate slice displacements. Slice‐following was feasible in all subjects, yielding physiologically accurate mitral flow patterns. In healthy subjects, mitral and aortic stroke volumes agreed, with ICC = 0.72 and 0.90 for static and slice‐following planes; with bias ±1 SDs 23.2 ± 13.2 mls and 8.4 ± 10.8 mls, respectively. Agreement with planimetry was stronger, with ICC = 0.84 and 0.96; bias ±1 SDs 13.7 ± 13.7 mls and –2.0 ± 8.8 mls for static and slice‐following planes, respectively.

**Data Conclusion:**

Slice‐following outperformed the conventional sequence and improved the accuracy of transmitral flow, which is important for assessment of diastolic function and mitral regurgitation.

**Level of Evidence:** 2

**Technical Efficacy:** Stage 2

J. Magn. Reson. Imaging 2020;51:1412–1421.

VALVULAR HEART DISEASE has a high and increasing prevalence, and is associated with worse outcome and heart failure.^1^, ^2^, ^3^ Mitral valve insufficiency or regurgitation is the most common valvular disease, and is often treated surgically.^2^, ^4^ Accurate transmitral flow measurements are of clinical importance before and after surgery.^5^, ^6^, ^7^ Another need for transmitral flow lies in the importance for evaluating diastolic function, an awareness that has been increasing since the 1990s with echocardiography, where mitral flow parameters E and A are fundamental.^8^

Echocardiography is the primary tool for mitral examinations, but has noteworthy limitations and is inadequate in patients with poor acoustic windows.^5^, ^6^ Magnetic resonance (MR) phase contrast (PC) can measure flow.^9^ However, valvular imaging is challenging due to the substantial valve movement in the apical–basal direction,^10^, ^11^, ^12^ meaning that a short‐axis slice will not depict the same tissue in all phases. Thus, mitral flow cannot be measured directly, and in clinical practice mitral regurgitation is inferred using the difference in stroke volumes (SV) from planimetry of the left ventricular (LV) cavity and aortic flow imaging.^13^ This indirect method has reduced accuracy, as it contains measurement errors from two different techniques, and while it provides total regurgitant volume, it yields no flow pattern information.

In diastole the mitral valve moves, resulting in a passive transfer of blood volume from the left atria to the LV,^14^, ^15^, ^16^ meaning that some portion of the SV is due to valvular motion. Therefore, any flow quantification at the valve must be corrected relative to valvular velocity for accurate SV calculation^15^, ^17^ which affects the quantification of diastolic parameters.^16^

An early approach for valvular MR used prospectively gated slice‐following PC with spin labeling to determine valvular motion.^18^, ^19^ More recent work has suggested 4D‐flow with valve tracking^20^, ^21^, ^22^, ^23^, ^24^ and tagging for aortic valve visualization.^25^ However, prospectively gated sequences do not capture end diastole, 4D‐flow is time‐consuming, and tissue‐tagging methods are challenging to implement robustly.

In this current era of feature‐tracking, we propose a retrospectively gated PC sequence that obtains slice‐following by prospective updates of the imaging slice, in real‐time, based on offline valvular feature‐tracking.^26^ This method also enables correction for valvular through‐plane motion. We hypothesized that transmitral flow can be measured more accurately with slice‐following compared to a static imaging plane. Therefore, this study aimed to implement such a sequence, validate its slice‐following functionality in a phantom and healthy subjects, and test its feasibility in patients with mitral valve dysfunction.

## Materials and Methods

### *Subjects*

This prospective study enrolled 16 healthy subjects with mean age ± standard deviation (SD) 33 ± 13 years (eight females, age 37 ± 17 years; eight males, age 30 ± 6 years) at either 1.5T Aera (*n* = 10) or 3T Prisma (*n* = 6) (Siemens, Erlangen, Germany) at two sites between January and September 2019. Additionally, 10 consecutive patients aged 60 ± 17 years (seven males, age 60 ± 18 years; three females, 59 ± 17 years) referred for cardiac MRI were included at 1.5T. Patients had findings of mitral regurgitation. All imaging was performed with Institutional Review Board (IRB) approval and all study participants provided written informed consent.

### *Healthy Subject Imaging*

**Figure 1:**
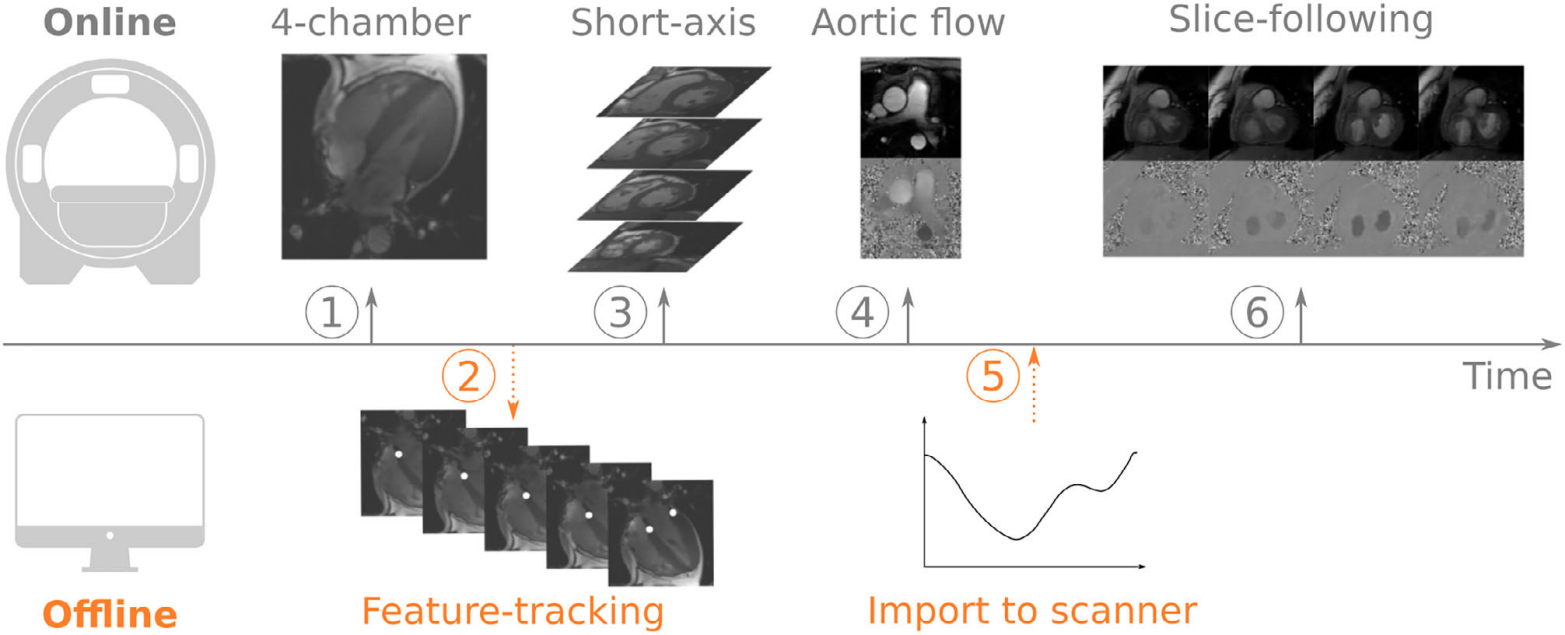
Workflow to obtain slice‐following phase contrast. 1) Obtain 4‐chamber cine. 2) Export the 4‐chamber cine offline and perform feature‐tracking of the mitral valve insertion points using Segment software. 3–4) While the offline analysis is being performed, continue scanning short‐axis cine stack and aortic flow. 5) Import the tracked displacements into the phase contrast slice‐following sequence. 6) Run the slice‐following sequence. The required time between the acquisition of the 4‐chamber cine and the slice‐following sequence was 5–10 minutes during which other sequences can be acquired.

Figure 1 illustrates a schematic overview of the MR protocol, consisting of 4‐chamber and short‐axis cine, aortic flow, and mitral flow imaging.

Cine imaging was performed with retrospectively ECG‐gated balanced steady‐state free‐precession (SSFP) at end‐expiration. Typical parameters: repetition time/echo time (TR/TE)/θ = 3.1 msec / 1.5 msec / 65‐40°, 890 Hz/pixel, matrix size 256 × 192, field of view (FOV) 360 mm, voxel size 1.4 × 1.9 × 6–8 mm, 30 calculated phases.

Aortic flow images were acquired as in our clinical protocol with 2D gradient echo through‐plane PC, free breathing. Typical parameters: retrospective ECG‐gating, velocity encoding (VENC) = 180 cm/s, TR/TE/θ = 4.9 msec / 2.7 msec / 20°, three bipolar pairs per segment, matrix size 208 × 168, FOV 330 mm, 1 average, voxel size 1.6 × 2.0 × 5 mm, 35 calculated phases.

Mitral imaging was performed with retrospectively ECG‐gated gradient echo cine PC with one end‐expiratory breath‐hold. The retrospective flow sequence software was modified to support slice‐following. A text file imported into the scanner environment was used by the MR sequence to perform real‐time cardiac phase‐dependent translation of the acquired slice. The text file contains the desired slice‐translation values (in mm) for each phase.

The slice translation values (in mm) were provided by a text file imported to the scanner during the exam, after analysis of the valve displacement, for each phase. The slice translation was performed by graphically prescribing multiple parallel slices spaced 1 mm apart. To translate the slice, the acquired slice was modified in real‐time during each cardiac cycle. Typical parameters: VENC = 150 cm/s, TR/TE/θ = 5.1 msec / 4.0 msec / 15°, matrix size 224 × 208, FOV 350 mm, voxel size 1.5 × 1.5 × 8 mm, three bipolar pairs per phase. Calculated phases were set to the RR‐interval divided by 8 TR, typically 22. Breath‐hold duration was typically 25 sec.

The phasic change in imaging plane was determined by measuring the mitral valve displacement as described previously.^26^ Briefly, a 4‐chamber cine with temporal resolution matched to the PC sequence was exported to an offline computer for feature‐tracking with manual corrections as needed. The subject‐specific mitral valve displacement was calculated at each phase as the average displacement of the septal and lateral mitral annular insertion points, and saved to a text file accessible by the pulse sequence.

**Figure 2:**
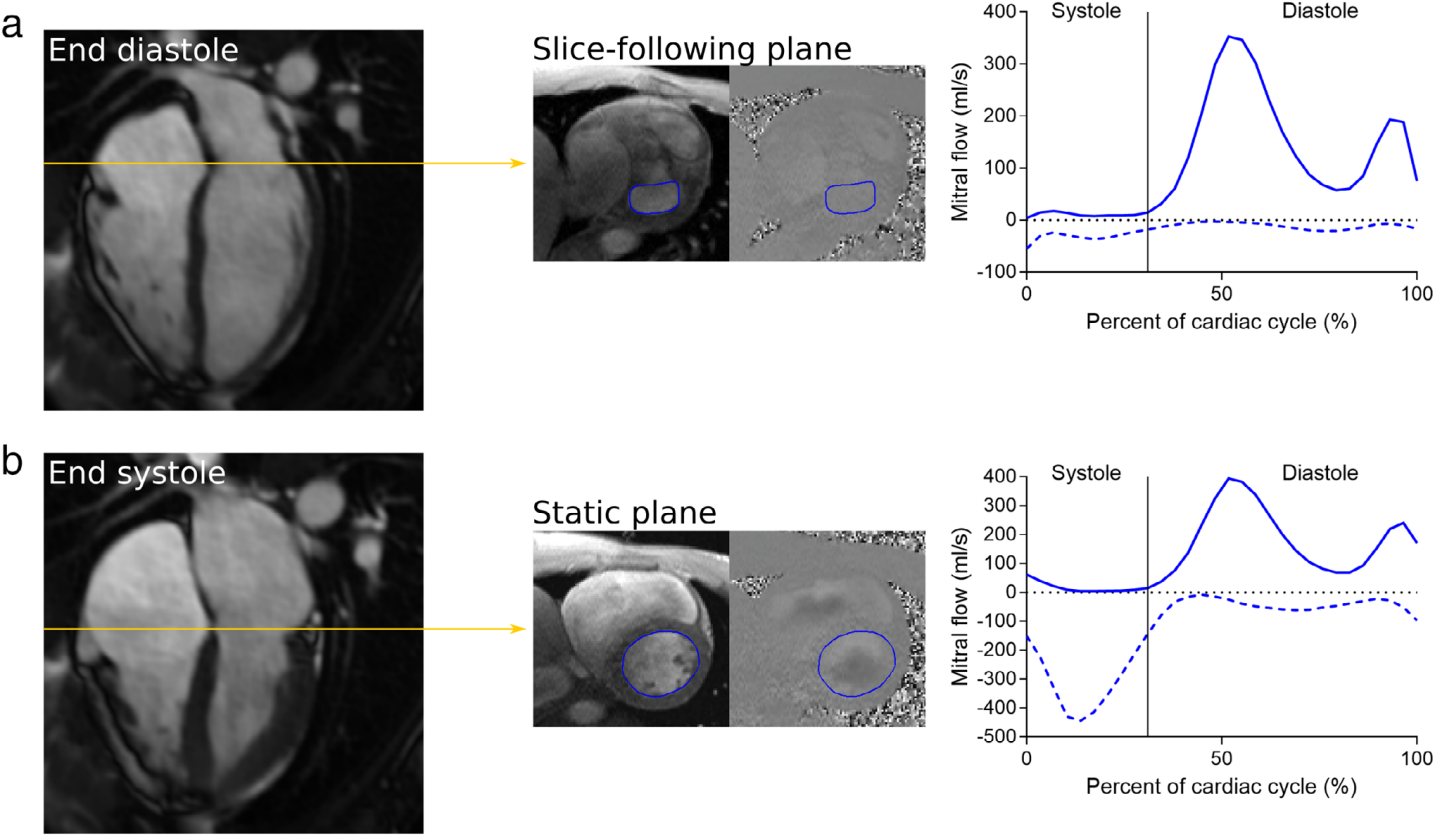
Illustration of the mitral valve image planes and the resulting data. Yellow lines represent the planned slice locations in the 4‐chamber long‐axis. Regions of interest for flow analysis along the mitral valvular borders are marked in blue in the phase contrast images, which are shown at end diastole. Mean forward and backward flow curves of all healthy subjects at their corresponding slice positions are shown as solid and dashed blue lines, respectively. Motion correction and background phase offset error correction was performed in all flow curves. **a:** Slice‐following phase contrast images were planned at the location of the mitral valve in the 4‐chamber long‐axis at end diastole. **b:** Conventional static phase contrast images were planned at the valve location in end systole.

Two mitral imaging planes were investigated: one slice‐following plane, and one static slice‐plane placed at the valve location in end systole (Fig. 2). The position of the mitral valve location in all long‐axis views were taken into account when planning the mitral imaging planes at the scanner.

### *Patient Imaging*

Patients were imaged in conjunction with their clinical scan. Slice‐following PC was imaged with spatial resolution 2.2 × 2.2 × 8 mm, which shortened the breath‐hold to 17 sec.

### *Phantom Imaging*

**Figure 3:**
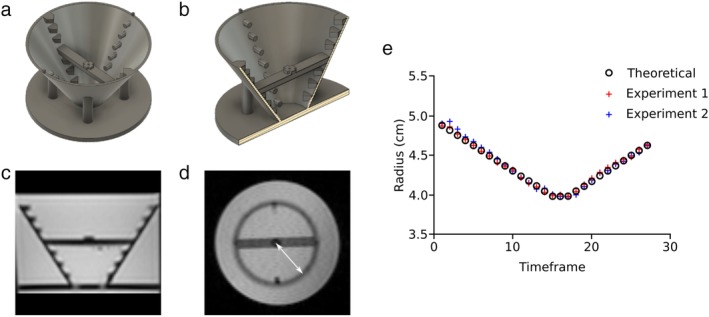
Phantom setup to confirm slice‐following. Cone geometry was 7 cm in height, top diameter was 12 cm, and bottom diameter was 3 cm. A 5‐mm thick horizontal bar was located midway at the height 3.5 cm, where the cone diameter was 7.5 cm. The center of the cone in both height and width was marked with an elevated cross on top of the bar. **a:** 3D rendering of the phantom cone from above. **b:** 3D rendering of the phantom cut through in the long‐axis direction. **c:** Long‐axis magnetic resonance image of the phantom. **d:** Short‐axis magnetic resonance image of the phantom at the middle of the cone. White line illustrates the radius in the depicted slice. **e:** Theoretical and measured cone radius in two phantom experiments, demonstrating accurate slice‐following.

The shift in slice position was validated at both sites using a motionless 3D printed cone shaped phantom (Fig. 3a–d) with a known analytical expression of radius as a function of height. The phantom was placed in a water‐filled container doped with gadolinium and subsequently scanned with the slice‐following sequence in a short‐axis view. Scan parameters were: 2D gradient echo cine PC, VENC = 100 cm/s, TR/TE/θ = 36.12 msec / 4.0 msec /15°, matrix size 128 × 128, FOV 250 mm, voxel size 1.95 × 1.95 × 8 mm, four bipolar pairs per phase and 27 calculated phases. Triggering was controlled with a scanner generated ECG‐signal, with an RR‐interval of 1000 msec. No feature‐tracking was performed, as the phantom was static. Instead, slice positioning was programmed to move in increments of 1 mm per phase, starting at the planned slice location, moving inferiorly for 15 phases, remaining stationary for three phases, and then moving superiorly for the remaining 10 phases. Slice position was then validated by measuring the depicted slice radius and comparing it with the theoretical radius at the programmed slice locations.

### *Image Analysis*

Image analysis was performed by F.S. (6 years of experience) using the freely available software Segment 2.2R6410 (Medviso, Lund, Sweden).^27^ For measuring planimetric SV, endocardial borders were delineated at end diastole and end systole in the contiguous SSFP short axis slices of the LV. Aortic root contours were defined semiautomatically for aortic flow measurements.^28^ Transmitral flow was assessed by drawing regions of interest along the mitral valvular borders. The LV outflow tract was not included in the regions of interest when visible.

The passively transferred blood volume is not accelerated over the valve and will therefore not be detected as flow in the PC images, since it is the valve itself that is moving. For accurate flow quantification, through‐plane motion correction was performed by subtracting mitral valve velocity from PC velocities at each phase in static and slice‐following images.^17^ Mitral valve velocity was calculated as the time‐derivative of the feature‐tracked displacement.^29^ Background phase offset error correction was performed in Segment by indicating regions of static tissue, which were time‐dependent for slice‐following images where the chest wall depiction varies with cardiac phase.

The angle between the planes connecting the tracked mitral insertion points at end diastole and end systole was calculated.

### *Quantitative Flow*

**Figure 4:**
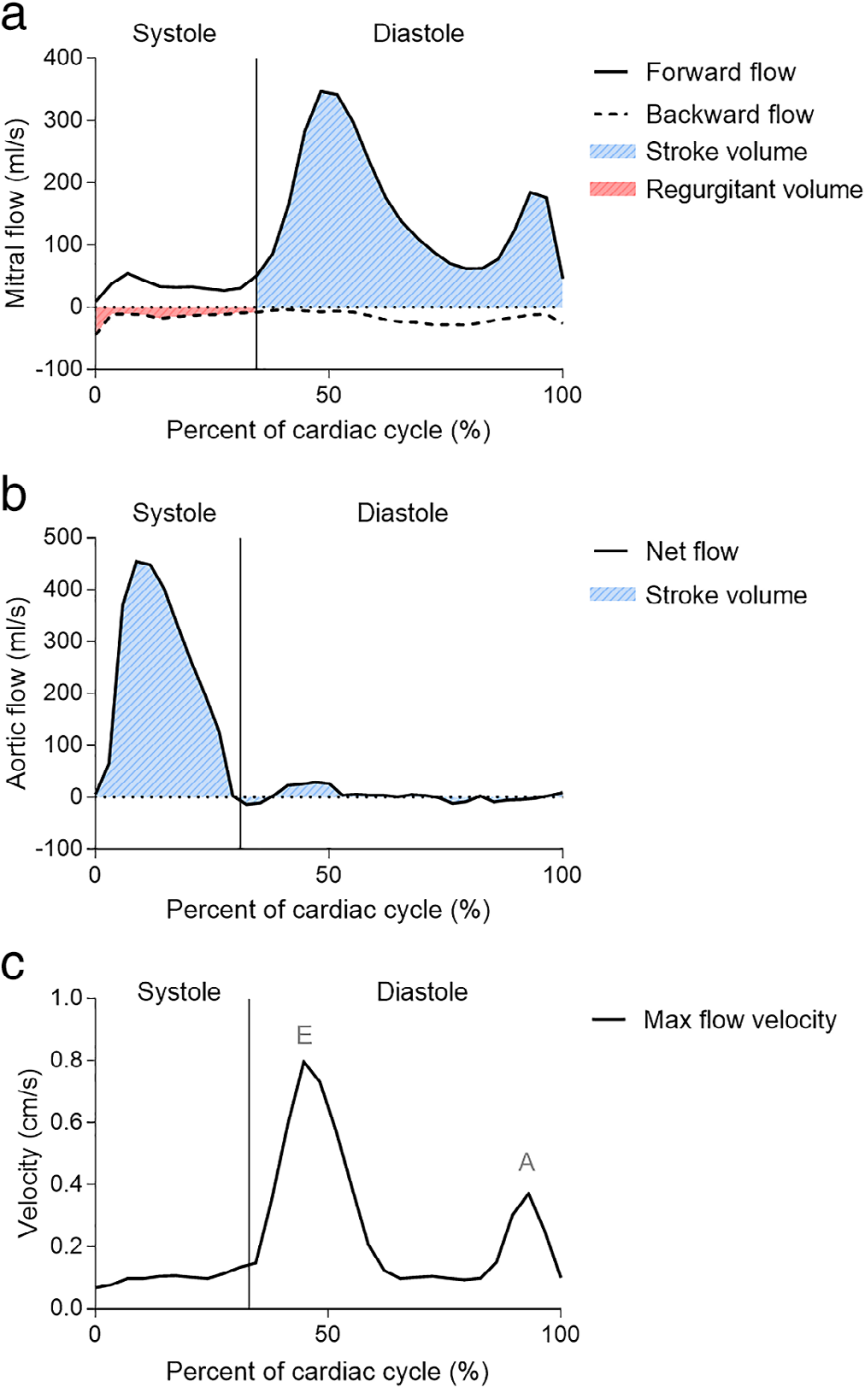
Illustration of quantitative flow. **a:** Mitral stroke volume was defined as the total volume of the forward flow during diastole. Mitral regurgitant volume was defined as the backward systolic flow. **b:** Aortic stroke volume was defined as the total volume of the net flow over the whole cardiac cycle. **c:** Diastolic parameters E and A were defined as the maximum mitral blood flow velocity at early diastole and atrial contraction, respectively.

Mitral SV was defined as the transmitral diastolic forward flow volume. Aortic SV was defined as the total net flow volume in the aortic root^13^ (Fig. 4). Mitral SV was compared to aortic and planimetric SV. Mitral regurgitant volume was quantified in three ways; 1) according to current guidelines as the difference of planimetric and aortic SV,^13^ 2) directly from the mitral images as the systolic backward flow volume, and 3) as the difference of mitral and aortic SV. The regurgitant fraction was defined as mitral regurgitant volume as a percentage of planimetric and mitral SV, respectively. End systole and end diastole were defined visually.

### *Diastolic Parameters*

Diastolic E and A were quantified without motion correction from the 95^th^ percentile of maximum velocity in early and late diastole (Fig. 4) and E/A was calculated.

### *Mitral Flow Patterns*

Forward, backward, and net transmitral flow curves were studied.

### *Observer Variability*

Interobserver and intraobserver variability analysis of slice‐following mitral SV was investigated in 10 subjects.

### *Statistical Analysis*

Statistical analysis was performed in GraphPad Prism 7.03 (La Jolla, CA). Linear regression, Pearson's R, parametric paired *t*‐tests, and Bland–Altman analysis were calculated with statistical significance for *P* < 0.05. Agreement between methods was assessed with ICC and interpreted as poor (ICC = 0.0–0.3), weak (ICC = 0.31–0.50), moderate (ICC = 0.51–0.70), strong (ICC = 0.71–0.90), or excellent (ICC = 0.91–1.00).^30^

## Results

**Figure 5:**
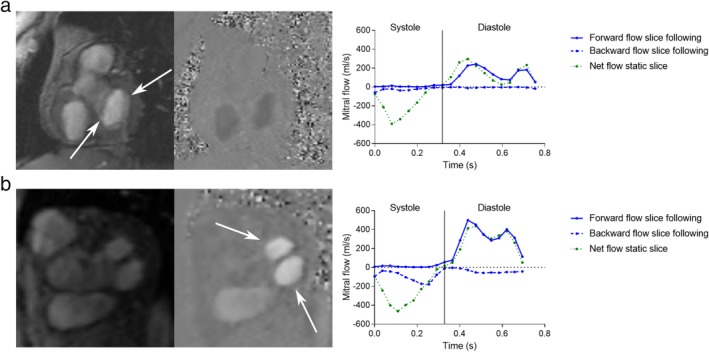
Example of mitral valve images acquired with valvular slice‐following phase contrast and corresponding flow profiles from motion‐corrected slice‐following (blue) and static images (green). **a:** Healthy subject acquired at 3T. White arrows points at the open mitral leaflets. **b:** Patient with double orifice mitral valve and mitral regurgitation imaged at 1.5T. White arrows points at the double orifice during diastole. A pronounced mitral regurgitation can be seen in the slice‐following backward flow profile, but is not distinguishable from the left ventricular outflow measured in the static net flow profile.

Quantification of mitral flow was feasible in all healthy subjects and patients. Examples of acquired slice‐following PC images at both field strengths are shown in Fig. 5, where both the valve morphology and flow can be visualized in a healthy subject and in patients. The corresponding net flow curves are also shown. An animation illustrating the difference in slice‐following and static mitral valve images is available as an online Supportive Information. Maximum mitral valve displacement measured with feature‐tracking was 15 ± 3 mm in the healthy subjects and 11 ± 4 mm in patients. The angle between the end‐diastolic and end‐systolic mitral valve location was 3.2 ± 1.5° in healthy subjects and 2.8 ± 2.4° in patients. The offline analysis, from the export of the 4‐chamber to the import of the displacement curve, took 5–10 minutes in total. Aortic flow was not acquired in one subject for technical reasons.

### *Phantom Imaging*

Cone slice radius over time from the two phantom experiments are shown in Fig. 3e, disclosing a very low bias of –0.01 ± 0.03 mm between theoretical and measured radii.

### *Quantitative Mitral Flow*

**Figure 6:**
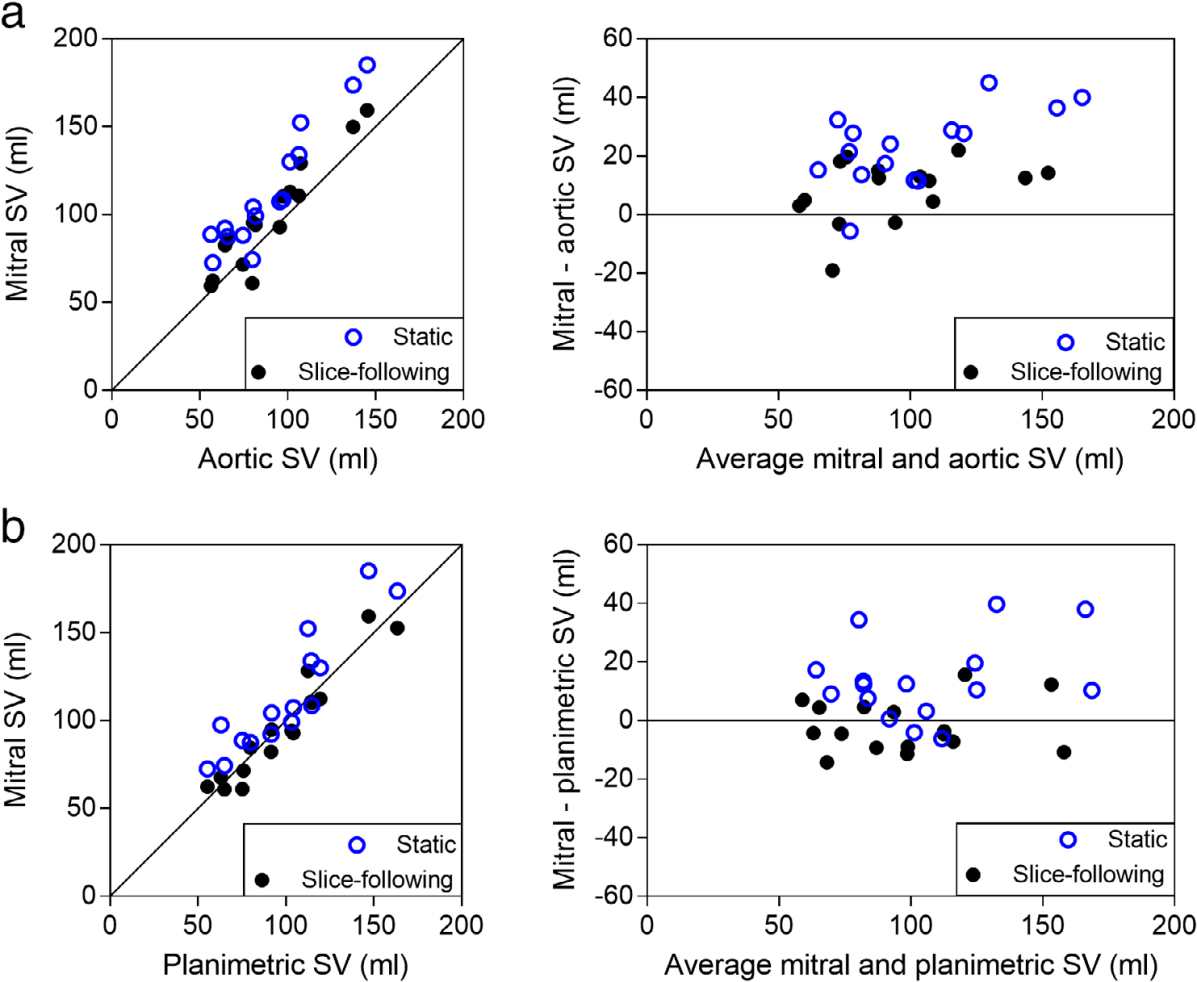
Comparison of SV in healthy subjects, showing scatterplots (left) and Bland–Altman analysis (right). Blue open circles show motion‐corrected static PC data and black closed circles shows motion‐corrected slice‐following data. **a:** Mitral SV with static and slice‐following PC vs. aortic SV. **b:** Mitral SV with static and slice‐following PC vs. planimetric SV.

Comparison of mitral SV to aortic and planimetric SV in healthy subjects is shown in Fig. 6 and Table 1. There was a strong agreement of mitral SV compared to aortic SV for both slice‐following and static imaging planes. Both imaging planes overestimated aortic SV, with a lower bias and SD for slice‐following than for static imaging. Part of this overestimation is explained by the ∼4% of the LV SV that flows into the coronary arteries, which is not accounted for in the aortic SV measurements due to the slice position of the aortic flow images in the ascending aorta. Agreement between mitral SV and planimetric SV was excellent using slice‐following and strong using static PC. Mitral SV yielded a slight underestimation of planimetric SV using slice‐following, and a larger overestimation using static PC. In these healthy subjects, strong agreement is expected, since mitral regurgitation is likely insignificant.

**Table 1:** Mitral Stroke Volume Assessment

	Mitral vs. Aortic SV		Mitral vs. Planimetric SV	
	Slice‐following	Static	Slice‐following	Static
ICC	0.90	0.72	0.96	0.84
Pearson R	0.94 (*P <* 0.0001)	0.94 (*P <* 0.0001)	0.96 (*P <* 0.0001)	0.91 (*P <* 0.0001)
Bias ± SD (ml)	8.4 ± 10.8	23.2 ± 13.1	–2.0 ± 8.8	13.7 ± 13.7
Limits of agreement (ml)	–12.6, 29.5	–2.4, 48.8	–19.3, 15.4	–13.2, 40.6

Agreement of mitral, aortic, and planimetric stroke volume (SV) in healthy subjects using motion corrected slice‐following and conventional static phase contrast (PC) images. ICC: Intraclass correlation coefficient; SD: standard deviation.

Even in the presence of mitral regurgitation, planimetric SV and mitral flow SV should be the same. Over all 26 subjects there was no difference between SV by planimetry and by slice‐following PC (99 ± 36 ml vs. 101 ± 43 ml, *P* = 0.3593), but with static PC, the difference was significant (99 ± 36 ml vs. 115 ± 48 ml, *P* = 0.0003). Without velocity correction of the valvular through‐plane motion, differences between planimetric and mitral SV using slice‐following PC were significant (99 ± 36 ml vs. 87 ± 38 ml, *P* = 0.0001). Hence, ∼14% of the blood volume that filled the LV was passively transferred through valvular motion, demonstrating the significance of this source of LV filling.

Transmitral net, forward, and backward flow volume for slice‐following and static images in healthy subjects are summarized in Table 2, with and without motion correction, in both systole and diastole. The motion‐corrected systolic forward flow was closer to zero using slice‐following compared to static images (*P* = 0.0034) in healthy subjects. The relationship of stroke volumes is plotted in Fig. 6; but Table 2 shows additional measurements that were analyzed. In particular, Table 2 shows that static PC, while reasonably accurate in estimating SV, cannot directly estimate regurgitant volume.

**Table 2:** Transmitral Flow Quantification

	Systolic flow volume (ml)			Diastolic flow volume (ml)		
	Net	Forward	Backward (mitral regurgitant volume)	Net	Forward (mitral SV)	Backward
Slice‐following	–5 ± 3	6 ± 3	–11 ± 4	91 ± 30	97 ± 31	–6 ± 4
Slice‐following, uncorrected	6 ± 3	11 ± 3	–6 ± 3	75 ± 27	83 ± 27	–8 ± 5
Static	–86 ± 26	8 ± 3	–95 ± 28	89 ± 24	112 ± 34	–23 ± 12
Static, uncorrected	–65 ± 21	14 ± 4	–79 ± 24	69 ± 18	97 ± 29	–28 ± 13

Transmitral flow volumes in healthy subjects as mean ± standard deviation quantified from slice‐following and conventional static phase contrast images in systole, diastole, and over the whole cardiac cycle. Mitral stroke volume (SV) was calculated as the forward diastolic flow volume, since left ventricular filling is achieved when the valve is open. Diastolic backward flow was assumed to reflect either ventricular or atrial flow rather than transmitral flow, and thus not accounted for in the mitral SV calculation. Systolic backward flow volume was considered the mitral regurgitant volume.

Mitral regurgitant volumes measured in patients using slice‐following PC compared to the conventional MR method (the difference in planimetric and aortic SV) are shown in Table 3 and in the Supporting Information 2. There was a strong agreement reflected by ICC and Pearson's correlation for both direct measurements and in the difference of mitral and aortic SV, but the limits of agreement were large. Regurgitant volumes were not measured in the static PC images since it is not possible to directly distinguish if the negative systolic flow in the measured flow curves corresponds to blood being accelerated towards the LV outflow or over the mitral valve towards the left atrium (see Table 2 and Fig. 5).

**Table 3:** Mitral Regurgitant Volumes

	Direct measurement vs. current guidelines	Mitral – aortic SV vs. current guidelines
ICC	0.75	0.85
Pearson R	0.86 (*P* = 0.0014)	0.95 (*P* < 0.0001)
Bias ± SD (ml)	–1.0 ± 17.8	8.6 ± 20.8
Limits of agreement (ml)	–36.0, 33.9	–32.1, 49.33

Agreement of mitral regurgitant volumes in patients. Quantification and according to current guidelines as the difference in planimetric and aortic stroke volume (SV) were compared to direct measurements as the backward systolic flow in motion corrected slice‐following phase contrast images, and as the difference in mitral and aortic stroke volume. ICC: Intraclass correlation coefficient; SD: standard deviation.

Quantified planimetric, aortic, and mitral SV as well as the measured mitral regurgitant volumes and fractions are summarized in Supporting Table 1 for healthy subjects and patients.

### *Diastolic Parameters*

Comparison of the diastolic parameters E and A and E/A for slice‐following vs. static PC in all subjects are shown in Table 4, calculated without motion correction, in analogy with echocardiography. The comparisons disclosed 10% differences between slice‐following and static imaging planes for E and A, but not E/A.

**Table 4:** Diastolic Parameters

	Slice‐following	Static	*P*‐value
E (m/s)	0.56 ± 0.17	0.65 ± 0.15	0.0002
A (m/s)	0.39 ± 0.12	0.48 ± 0.19	0.001
E/A	1.6 ± 1.0	1. 7 ± 1.0	0.4969

Diastolic parameters E, A, and E/A as mean ± SD quantified from slice‐following phase contrast (PC) and conventional static PC images in healthy subjects and patients. All parameters were calculated without motion correction, in analogy to echocardiography. Differences in slice‐following and static images were investigated with a paired parametric *t*‐test.

### *Mitral Flow Patterns*

Mitral flow patterns were in line with what is physiologically expected, clearly showing diastolic E and A waves. Flow profiles for the acquired images in the healthy controls are shown in Fig. 2, averaged over all subjects. The overall flow patterns were similar in diastole, but disclosed differences in systole. Systolic flow patterns of slice‐following PC were close to zero in healthy subjects, and exhibited systolic backward flow in patients with mitral regurgitation. With static PC, flow patterns always exhibited systolic backward flow, reflecting flow towards the LV outflow tract.

### *Observer Variability*

Intraobserver variability of mitral SV measurements in the valvular slice‐following image plane disclosed an excellent level of agreement, with ICC = 0.99 and low bias of –0.9 ± 3.3 ml. The level of agreement in the interobserver variability was also excellent, with ICC = 0.98 and a low bias of 3.9 ± 3.8 ml.

## Discussion

This study presents a highly feasible retrospectively ECG‐gated slice‐following PC MR sequence with offline feature‐tracking, by which transmitral flow can be directly measured. Our major finding is that mitral SV was more accurately quantified with slice‐following PC compared to conventional static PC, demonstrated by smaller discrepancies with aortic and planimetric SV in healthy young subjects. Slice‐following PC also provided physiologically accurate flow wave forms, and the ability to measure mitral regurgitant flow as backward systolic flow.

Kozerke et al introduced the slice‐following PC concept and studied regurgitation in three mitral valve patients, but did not compare aortic and mitral SV.^18^, ^19^ That method used spin labeling to track the slice, which differs from the feature‐tracking approach shown here. Westenberg et al showed that slice‐following in postprocessing of 4D‐flow improved mitral SV compared to static PC.^20^

Calculation of aortic SV as net flow over the heartbeat is well established and based on physiology.^13^ Aortic flow is measured above the valve location in the ascending aorta, and therefore part of the SV will not pass the imaging plane until diastole, mainly due to the aortic valve through‐plane motion towards the base.^18^ Furthermore, the aortic valve closes due to backflow of blood from the ascending aorta towards the valve, justifying the accounting of negative flow in SV quantification. In contrast, the definition of mitral SV is less established and previous slice‐following studies used either diastolic inflow^19^ or net flow over the heartbeat.^20^

The division of systolic and diastolic flow, however, is important when studying mitral flow. Since the mitral valve is closed during systole, there is no pronounced flow in this phase unless the valve is insufficient, in which case there is a regurgitant backward flow. In diastole, the open valve allows for LV filling as a combination of blood flow and volume transfer due to the valve movement. Hence, only diastolic flow was used in our mitral SV measurements. Furthermore, diastolic backward flow was not assumed to flow into the LA, but is probably reflecting either ventricular or atrial flow, e.g. part of the vortex ring at the inflow tract.^31^

Using these definitions, motion‐corrected slice‐following PC agreed more strongly with planimetry and aortic flow, compared to static PC, in the measurement of SV. The higher accuracy using slice‐following was also reflected in the systolic flow curve patterns. Regurgitant volume could be measured directly with slice‐following, but this was impossible for static PC. In subjects with mitral regurgitation, negative transmitral flow was distinguishable during systole. In healthy subjects with nonexistent or low degrees of regurgitation, systolic flow volumes were close to zero. In contrast, static slices disclosed substantial negative systolic flows that are explained by the LV outflow tract. Thus, the improved accuracy of transmitral flow using slice‐following was evident in both quantitative measures and in the flow patterns.

Valvular through‐plane motion correction enabled the accounting of passively transferred blood from the atria to the ventricle in diastole, improving the agreement of slice‐following PC mitral inflow with planimetric SV, confirming previous work by Carlhäll et al.^15^

Echocardiographic parameters E and A have been shown to agree with corresponding cardiac MR measurements.^29^, ^32^ In the current study, we found that E and A values were lower using slice‐following than static planes, while the E/A ratio was not different. Higher velocities measured by the static PC approach are expected, since the slice planes were different. The slice‐following plane was located at the level of the valve throughout the cardiac cycle. The static plane was closer to the open mitral valve tips in diastole, when E and A were measured. All fluid streams have a convergence zone downstream of the inflow orifice called the vena contracta, the point where the narrowest jet diameter and highest flow velocity materializes.^33^ Therefore, E and A were expected to be higher below the valve plane, and the finding confirms previous findings by Calkoen et al.^22^ Considering that echocardiographic E and A measurements are assessed at the valve tips and has a higher temporal resolution compared to MR, they should not be directly compared to the corresponding values obtained with slice‐following. Further studies of E and A could compare an optimized MR approach, with respect to slice plane and temporal resolution, to echocardiography, the current gold standard.

Our study had several limitations. As long‐axis and slice‐following images were acquired 5–20 minutes apart and the breath‐holds for slice‐following PC were long, different breath‐hold positions or patient movement in this period of time could make the valvular displacements inexact. Furthermore, a large heart rate variation might impact the duration of diastasis.^34^ However, in our experience valve motion is rather consistent and should therefore only impart a minor reduction in slice‐following accuracy. Chest wall artifacts appear in slice‐following PC, and are especially evident for large displacements between two phases, because the magnetization's steady state is perturbed. However, these artifacts did not overlie the mitral valve. Mitral and planimetric SV were measured from images acquired at breath‐hold, while aortic flow was imaged at free breathing. Hence, agreement between mitral and aortic SV could potentially be improved by matching respiratory states. Moreover, direct quantification of regurgitant volume was not fully studied in our cohort, where patients only had a mild or moderate degree of mitral regurgitation. Studies in a larger group of subjects with regurgitation are warranted to establish the robustness of the proposed method, especially for direct quantification of regurgitant mitral flow. Accurate measurement of the regurgitant fraction using a slice‐following PC approach during systole might be affected by turbulence and angulation of regurgitant jet,^23^, ^35^ which can cause errors in flow due to phase‐dispersion and partial volume effects.^36^ While the regurgitant jet is often angulated and turbulent, we found the mitral inflow to be reasonably through‐plane directed over the cardiac cycle, which provides the opportunity to calculate flow based on two phase‐contrast scans (aortic flow–mitral valve flow). Finally, future work for our PC implementation to gain clinical applicability includes increasing the temporal resolution and further speed‐ups or a free‐breathing approach that might improve the feasibility and quality.

In conclusion, a slice‐following PC sequence that follows the mitral valve throughout the heartbeat has been developed. The method outperforms conventional static imaging in accurately quantifying mitral diastolic and, especially, systolic flow. Hence, the sequence is a promising method for improving the accuracy of transvalvular flow using MR.

## Supporting information


**Supporting Information 1** Animated illustration of slice‐following and static image planes. The slice‐following image marked in yellow is moving to depict a valvular plane in all phases. In contrast, the static image plane marked in white was planned at the valve location in end systole and remains in the same spatial location over the heartbeat.
**Supporting Information 2.** Mitral regurgitant volumes (top row) and fractions (bottom row) calculated using systolic backward (bwd) flow and the difference in mitral and aortic stroke volume (SV), compared with current guidelines as the difference in planimetric and aortic SV. A) Mitral regurgitant volume measured as systolic backward flow. B) Mitral regurgitant volume measured as the difference in mitral and aortic SV. C) Mitral regurgitant fraction measured with systolic backward flow. D) Mitral regurgitant fraction measured with the difference in mitral and aortic SV.
**Supporting Table 1**.

**Video S1**
