# Supplementary material for: Valvular imaging in the era of feature‐tracking: A slice‐following cardiac MR sequence to measure mitral flow
Source: J Magn Reson Imaging. 2019 Oct 25;51(5):1412–21. doi: 10.1002/jmri.26971 (PMC7217167; doi:10.1002/jmri.26971)
Supplement: Supplementary file 1 — Supporting Information 1 Animated illustration of slice‐following and static image planes. The slice‐following image marked in yellow is moving to depict a valvular plane in all phases. In contrast, the static image plane marked in white was planned at the valve location in end systole and remains in the same spatial location over the heartbeat. Supporting Information 2. Mitral regurgitant volumes (top row) and fractions (bottom row) calculated using systolic backward (bwd) flow and the difference in mitral and aortic stroke volume (SV), compared with current guidelines as the difference in planimetric and aortic SV. A) Mitral regurgitant volume measured as systolic backward flow. B) Mitral regurgitant volume measured as the difference in mitral and aortic SV. C) Mitral regurgitant fraction measured with systolic backward flow. D) Mitral regurgitant fraction measured with the difference in mitral and aortic SV. Supporting Table 1. [file JMRI-51-1412-s001.docx]

**Supporting Information**

**
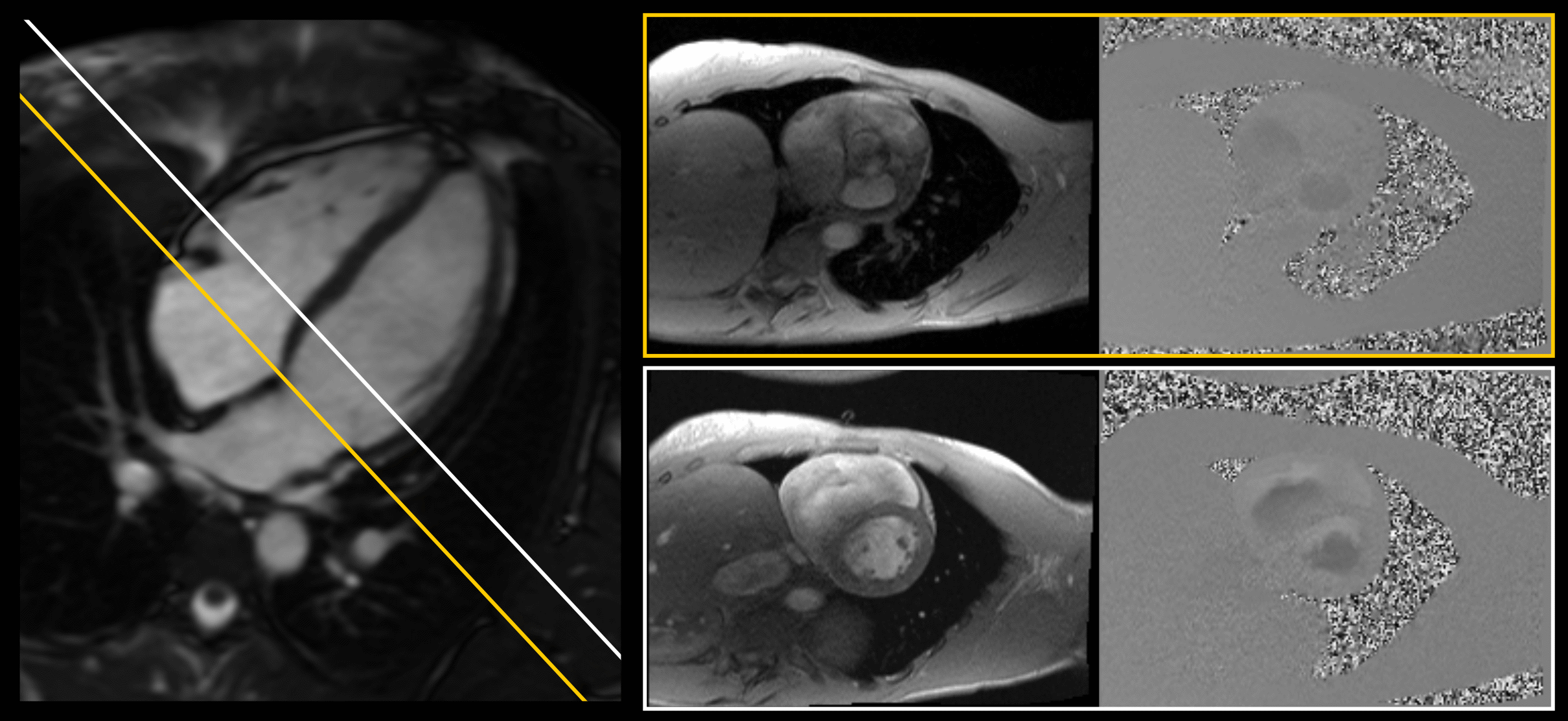
**

**Supporting Information 1.** Animated illustration of slice-following and static image planes. The slice-following image marked in yellow is moving to depict a valvular plane in all phases. In contrast, the static image plane marked in white was planned at the valve location in end-systole and remains in the same spatial location over the heartbeat.

**
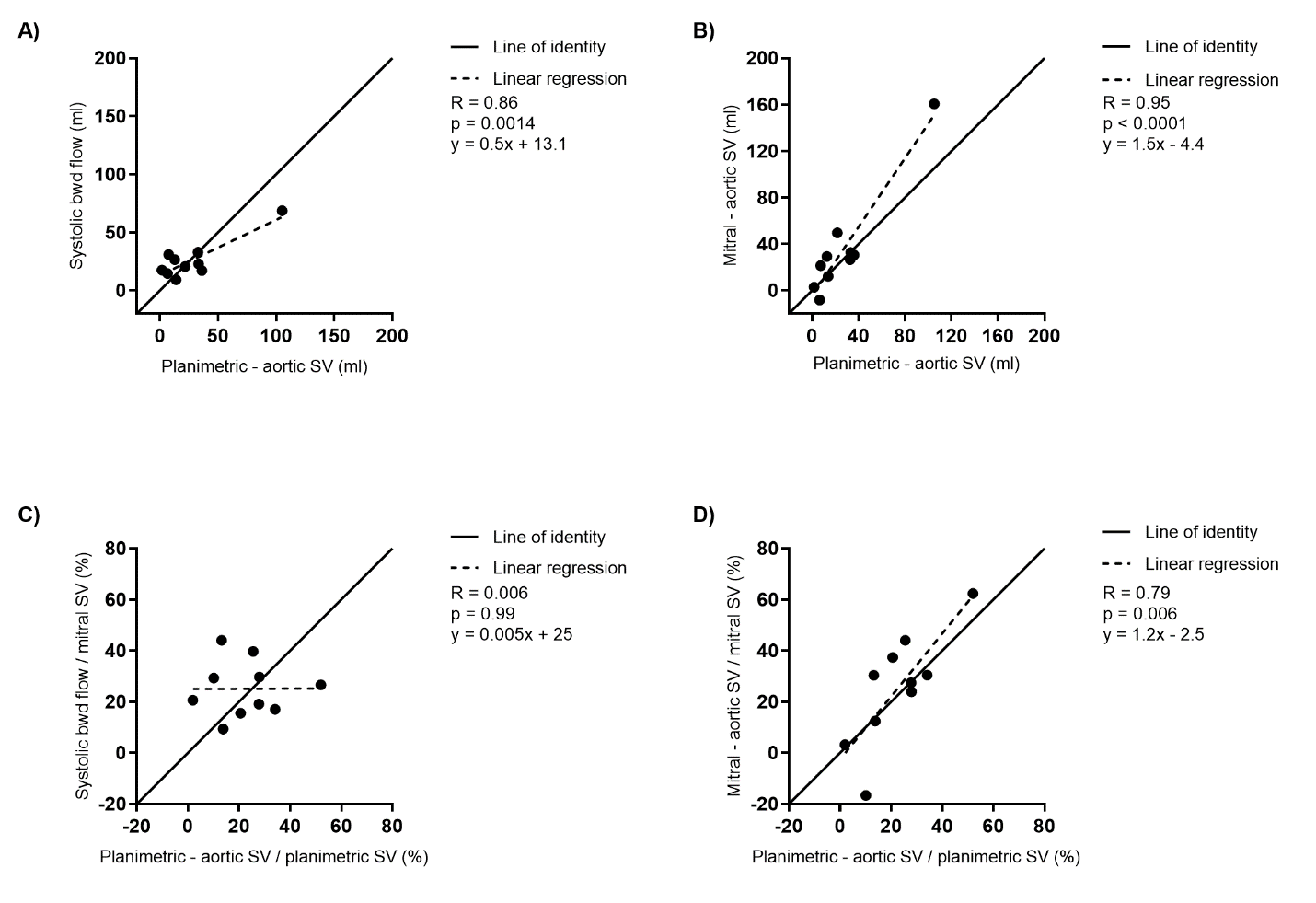
**

**Supporting Information 2.** Mitral regurgitant volumes (top row) and fractions (bottom row) calculated using systolic backward (bwd) flow and the difference in mitral and aortic stroke volume (SV), compared with current guidelines as the difference in planimetric and aortic SV. A) Mitral regurgitant volume measured as systolic backward flow. B) Mitral regurgitant volume measured as the difference in mitral and aortic SV. C) Mitral regurgitant fraction measured with systolic backward flow. D) Mitral regurgitant fraction measured with the difference in mitral and aortic SV.

**Supporting Table 1.**

|  | Healthy subjects | Patients |
| --- | --- | --- |
| Planimetric SV (ml) | 99±30 | 100±44 |
| Aortic SV (ml) | 90±27 | 73±19 |
| Mitral SV, slice-following (ml) | 97±31 | 109±58 |
| Mitral SV, static (ml) | 112±34 | 120±67 |
| Mitral regurgitant volume, planimetric – aortic SV (ml) | 12±9 | 27±30 |
| Mitral regurgitant fraction, planimetric – aortic SV (%) | 12±9 | 23±14 |
| Mitral regurgitant volume, direct slice-following (ml) | 11±4 | 25±17 |
| Mitral regurgitant fraction, slice-following (%) | 11±4 | 26±17 |
| Mitral regurgitant volume, mitral – aortic SV (ml) | 10±8 | 36±47 |
| Mitral regurgitant fraction, mitral – aortic SV (%) | 9±8 | 25±22 |

Quantification of stroke volume (SV), mitral regurgitant volume and mitral regurgitant fraction as mean ± standard deviation quantified by different methods in healthy subjects and patients.
