# Supplementary figures and images for: Valvular imaging in the era of feature‐tracking: A slice‐following cardiac MR sequence to measure mitral flow
Source: J Magn Reson Imaging. 2019 Oct 25;51(5):1412–21. doi: 10.1002/jmri.26971 (PMC7217167; doi:10.1002/jmri.26971)

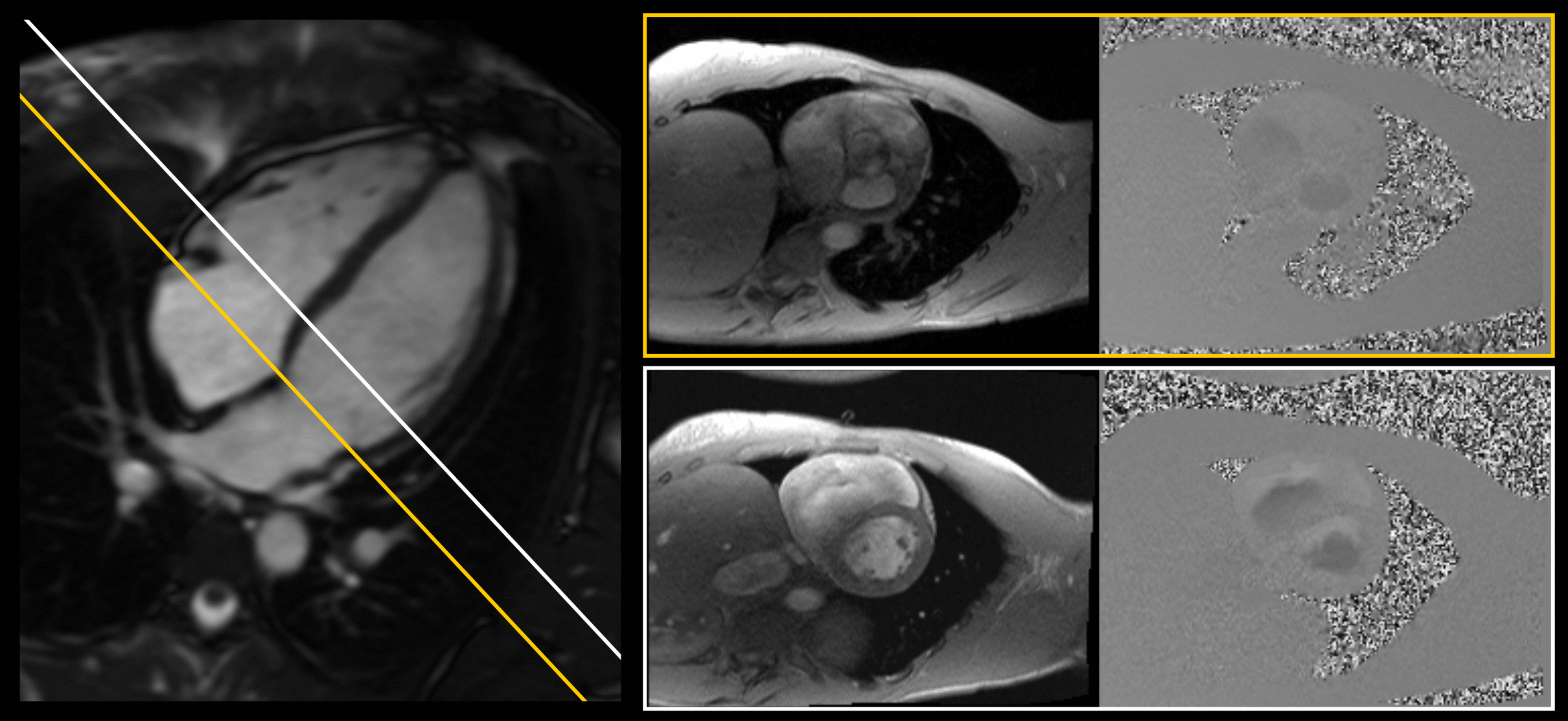

Supplement: Supplementary file 2 — Video S1 [file JMRI-51-1412-s002.gif]
